# Acute rhabdomyolysis and delayed pericardial effusion in an Italian patient with Ebola virus disease: a case report

**DOI:** 10.1186/s12879-017-2689-x

**Published:** 2017-08-30

**Authors:** Emanuele Nicastri, Antonio Brucato, Nicola Petrosillo, Gianluigi Biava, Timothy M. Uyeki, Giuseppe Ippolito, INMI’s Ebola Team, Nicola Petrosillo, Emanuele Nicastri, Nazario Bevilacqua, Evangelo Boumis, Pierangelo Chinello, Stefania Cicalini, Angela Corpolongo, Vincenzo Galati, Andrea Mariano, Silvia Rosati, Fabrizio Taglietti, Laura Vincenzi, Mario Antonini, Ilaria Caravella, Gabriele Garotto, Luisa Marchioni, Micaela Maritti, Pietro Balestra, Martina Ricottini, Elisa Busi Rizzi, Gianluigi Biava, Maria Rosaria Capobianchi, Antonino di Caro, Concetta Castilletti, Licia Bordi, Eleonora Lalle, Mirella Biava, Silvia Meschi, Daniele Lapa, Patrizia Marsella, Francesca Colavita, Roberta Chiappini, Antonio Mazzarelli, Serena Quartu, Chiara Agrati, Fabrizio Carletti, Federica Forbici, Maria Beatrice Valli, Isabella Abbate, Alessandra Amendola, Anna Rosa Garbuglia, Maria Grazia Paglia, Eugenio Bordi, Damiano Travaglini, Antonietta Toffoletti, Gianni Battisti, Alessanda Coppola, Loredana De Marchis, Nicola De Marco, Paolo Giacomini, Fabio Di Gianbattista, Mario Guiducci, Antonio Marasco, Antonella Marzolini, Alessandro Mercuri, Paola Nieddu, Silvia Ondedei, Maurizio Vescovo, Laura Vitolo, Maurizio Morea, Gaetano Battisti, Marco Liguori, Francesco Nicola Lauria, Vincenzo Puro, Antonio Russo, Paolo D’Aprile, Antonella Petrecchia, Marco Gentile, Silvia Pittalis, Lorena Martini, Francesco Maria Fusco, Simone Lanini, Andrea Antinori, Marina Cerimele, Giuseppe Ippolito, Marta Branca

**Affiliations:** 1 National Institute for Infectious Diseases – INMI - Lazzaro Spallanzani IRCCS, Via Portuense 292, 00149 Rome, Italy; 2 Internal Medicine Ospedale Papa Giovanni XXIII, Bergamo, Italy; 3 Centers for Disease Control and Prevention, Atlanta, GA USA

**Keywords:** Ebola Virus Disease, Rhabdomyolysis, Pericardial effusion

## Abstract

**Background:**

During the 2013–2016 West Africa Ebola virus disease (EVD) epidemic, some EVD patients, mostly health care workers, were evacuated to Europe and the USA.

**Case presentation:**

In May 2015, a 37-year old male nurse contracted Ebola virus disease in Sierra Leone. After Ebola virus detection in plasma, he was medically-evacuated to Italy. At admission, rhabdomyolysis was clinically and laboratory-diagnosed and was treated with aggressive hydration, oral favipiravir and intravenous investigational monoclonal antibodies against Ebola virus. The recovery clinical phase was complicated by a febrile thrombocytopenic syndrome with pericardial effusion treated with corticosteroids for 10 days and indomethacin for 2 months. No evidence of recurrence is reported.

**Conclusions:**

A febrile thrombocytopenic syndrome with pericardial effusion during the recovery phase of EVD appears to be uncommon. Clinical improvement with corticosteroid treatment suggests that an immune-mediated mechanism contributed to the pericardial effusion.

## Background

The 2013–6 West Africa Ebola virus disease (EVD) epidemic resulted in 28,616 confirmed, probable and suspected cases reported in Guinea, Liberia and Sierra Leone, with 11,310 deaths [1]. A small number of EVD cases were medically-evacuated or imported to Europe and the U.S., with limited secondary transmission in Spain and USA, in health care workers [2]. Pericardial involvement has rarely been reported in EVD patients [3–5]. Here we describe a case of acute rhabdomyolysis with delayed pericardial effusion in a nurse with EVD.

## Case presentation

In May 2015, a 37-year old male nurse who had been working in Sierra Leone was admitted to the Spallanzani Hospital, Rome, Italy for EVD clinical management. Medical, family and psychosocial history was non-contributory. Findings at admission, 3 days after symptom onset, included fever (39.0 °C), myalgia, conjunctivitis, diarrhoea, rhabdomyolysis [elevated serum creatine kinase (CK) level (785 IU/L, normal range 22–269)] with normal renal function, and Ebola virus (EBOV) load in plasma was 5 × 10^7^ copies/ml.

**Fig. 1:**
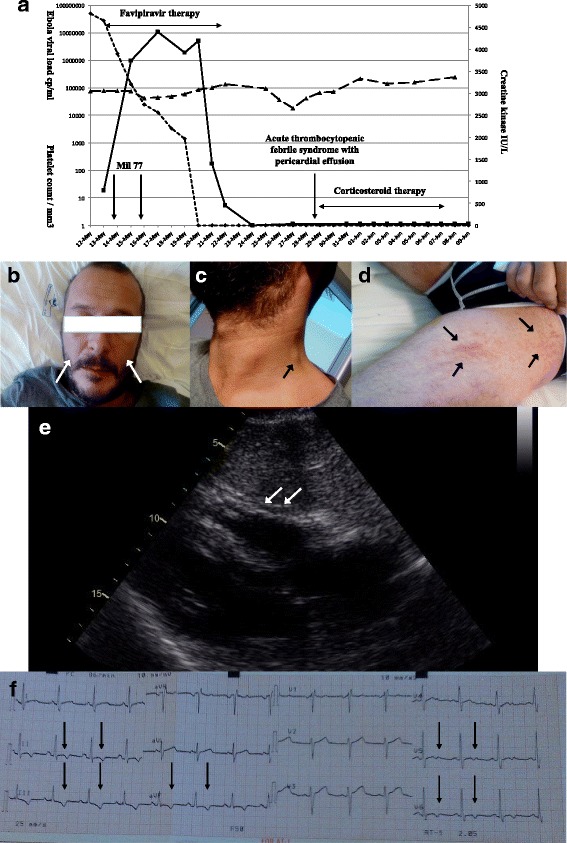
Ebola plasma viral load, creatine kinase levels, platelet count, timing of drug administration and of occurrence of the thrombocytopenic febrile syndrome (panel **a**); Skin lesions on the face and on the left thigh, and neck adenopathy (panels **b**-**d**); Echocardiographic evidence of MILD circumferential pericardial effusion at the time of the thrombocytopenic febrile syndrome and ECG showing ECG showed diffuse nonspecific abnormalities (panels **e** and **f**). Legend of panel A: The Y-axis indicates Ebola viral load (copies/ml) and platelet count (platelet/mm3). The Z-axis indicates creatine kinase levels (International Units/Liter). CK creatine kinase

Oral favipiravir (Toyama Chemical Co, Japan) was administered (6-g loading dose and 1200 mg twice daily for 10 days) [6, 7]. Two doses of investigational monoclonal antibodies against EBOV (MIL77, Mabworks Beijing China) were given (50 mg/kg IV) 3 days apart. Empiric antibiotic treatment with intravenous ceftriaxone (2 g daily) and oral levofloxacin (750 mg daily), and intravenous crystalloid solution, were administered daily with progressive clinical improvement. CK level peaked on illness day 5 (4400 IU/ml) and declined to normal on illness day 10 (Fig. 1a). Renal function remained normal. The plasma EBOV load was undetectable on day 11 (Fig. 1a).

On illness day 19, a febrile syndrome with diffuse adenopathy, confluent skin rash and marked thrombocytopenia (18,000/mm3) occurred (Fig. 1b-d). ECG showed diffuse nonspecific abnormalities in repolarisation, and an echocardiogram showed a mild circumferential pericardial effusion (largest echo-free space in tele-diastole <10 mm) (Fig. 1e-f). Chest pain and pericardial rub were absent. High-dose corticosteroid therapy was initiated with immediate clinical improvement; methylprednisolone, 1 g IV daily for 2 days, reduced to 500 mg on day 21 and 250 mg on day 22, and then switched to oral prednisone on day 23, with normalization of platelet count. Serum tested positive for rheumatoid factor, Waaler Rose, and circulating immune complexes. At discharge on illness day 29, a minimal pericardial effusion was present. Corticosteroid treatment was stopped and oral indomethacin 25 mg twice daily was prescribed. Echocardiographic examination performed 60 days after discharge showed complete resolution of the pericardial effusion and indomethacin therapy was stopped. There was no evidence of pericardial effusion at 18 month follow-up visit.

## Discussion and conclusions

A febrile thrombocytopenic syndrome with pericardial effusion during the recovery phase of EVD appears to be uncommon. Pericarditis was suggested as a cause of retrosternal pain in some patients and pericardial effusion was confirmed in one fatal EVD case during the 1995 Kikwit outbreak [3]. Pericardial effusion was reported in a critically ill EVD patient in Germany [4], and in two EVD patients in Guinea in 2014 [5].

Immune activation has been described in a small number of EVD patients [8]. In this case, EBOV infection may have triggered inflammation resulting in rhabdomyolysis, and after viremia resolved, prolonged immune activation may have caused pericardial tissue injury [9]. A serum-sickness disease induced by the monoclonal antibody against EBOV that was administered is another possible explanation [8]. Clinical improvement with corticosteroid treatment suggests that an immune-mediated mechanism likely contributed to the development of the pericardial effusion.

## Abbreviations

CK: Creatine kinase
EBOV: Ebola virus
EVD: Ebola virus disease
PCR: Polymerase chain reaction
